# A genome-wide assessment of conserved SNP alleles reveals a panel of regulatory SNPs relevant to the peripheral nerve

**DOI:** 10.1186/s12864-018-4692-z

**Published:** 2018-05-02

**Authors:** William D. Law, Elizabeth A. Fogarty, Aimée Vester, Anthony Antonellis

**Affiliations:** 1 Department of Human Genetics, University of Michigan Medical School, Ann Arbor, MI USA; 2 Neuroscience Graduate Program, University of Michigan Medical School, Ann Arbor, MI USA; 3 Department of Neurology, University of Michigan Medical School, 3710A Medical Sciences II, 1241 E. Catherine St. SPC 5618, Ann Arbor, MI 48109 USA

**Keywords:** SOX10, Schwann cells, Peripheral nerve, Transcriptional regulation, Neuropathy, TUBB2B, Enhancer

## Abstract

**Background:**

Identifying functional non-coding variation is critical for defining the genetic contributions to human disease. While single-nucleotide polymorphisms (SNPs) within *cis*-acting transcriptional regulatory elements have been implicated in disease pathogenesis, not all cell types have been assessed and functional validations have been limited. In particular, the cells of the peripheral nervous system have been excluded from genome-wide efforts to link non-coding SNPs to altered gene function. Addressing this gap is essential for defining the genetic architecture of diseases that affect the peripheral nerve. We developed a computational pipeline to identify SNPs that affect regulatory function (rSNPs) and evaluated our predictions on a set of 144 regions in Schwann cells, motor neurons, and muscle cells.

**Results:**

We identified 28 regions that display regulatory activity in at least one cell type and 13 SNPs that affect regulatory function. We then tailored our pipeline to one peripheral nerve cell type by incorporating SOX10 ChIP-Seq data; SOX10 is essential for Schwann cells. We prioritized 22 putative SOX10 response elements harboring a SNP and rapidly validated two rSNPs. We then selected one of these elements for further characterization to assess the biological relevance of our approach. Deletion of the element from the genome of cultured Schwann cells—followed by differential gene expression studies—revealed *Tubb2b* as a candidate target gene. Studying the enhancer in developing mouse embryos revealed activity in SOX10-positive cells including the dorsal root ganglia and melanoblasts.

**Conclusions:**

Our efforts provide insight into the utility of employing strict conservation for rSNP discovery. This strategy, combined with functional analyses, can yield candidate target genes. In support of this, our efforts suggest that investigating the role of *Tubb2b* in SOX10-positive cells may reveal novel biology within these cell populations.

**Electronic supplementary material:**

The online version of this article (10.1186/s12864-018-4692-z) contains supplementary material, which is available to authorized users.

## Background

The identification of functional genetic variation is critical for understanding the allelic, locus, and clinical heterogeneity of human inherited disease. Genome-wide association studies (GWAS) have identified common variants in non-coding sequences—including transcriptional regulatory elements—that have implications for defining disease-causing and disease-modifying variants [1]. A major challenge remains in understanding the functional consequences of the implicated variants, which are frequently single-nucleotide polymorphisms (SNPs). Predicting the functional effects of SNPs is difficult due to many factors including limited knowledge of *cis*-acting regulatory elements (CREs) and an incomplete vocabulary of transcription factor binding sites (TFBS), especially in understudied cell populations. Furthermore, it is difficult to predict the location of CREs as they may reside anywhere in the genome and millions of base pairs distal to the genes that they regulate [2]. While ENCODE improved our TFBS vocabulary and, as a consequence, our understanding of functional genetic variation, many tissue types have not been assessed and many of these predictions have not been functionally validated [3]. The ability to predict the effect of non-coding variation on gene expression and rapidly validate genomic regions for *cis*-regulatory activity will aid the identification of modifiers of human disease [4].

Charcot-Marie-Tooth (CMT) disease displays vast clinical heterogeneity and variable penetrance. CMT is an inherited peripheral neuropathy that affects 1 in 2500 individuals worldwide and is characterized by impaired motor and sensory nerve function in the distal extremities [5]. CMT is subdivided into two major classes: demyelinating (CMT1) and axonal (CMT2) [6]. CMT1 affects Schwann cell myelination and, predictably, genes associated with CMT1, including myelin protein zero (*MPZ*) [7, 8] and peripheral myelin protein 22 (*PMP22*) [9, 10], are critical for that process. Similarly, the genes implicated in CMT2, which affects motor and sensory axons, include neurofilament light chain (*NEFL*) [11] and mitofusin 2 (*MFN2*) [12], which are critical for axon function. Interestingly, while over 80 genes have been implicated in CMT disease [13], phenotypic variability is observed even among patients with a molecularly indistinguishable mutation. For example, patients with a duplication of *PMP22* have variable age of onset (3-73 years of age), variable motor and sensory nerve involvement, and display a broad spectrum of severity, ranging from mild difficulty in walking or running to impairment requiring a wheelchair [14].

Among genetic variants that may modify human disease phenotypes, regulatory SNPs (rSNPs) present a unique challenge. Single-base-pair changes within sequence space that lacks a vocabulary leave few clues to their influence. rSNPs typically reside within transcriptional regulatory elements and have allele-specific effects on gene expression. An rSNP may increase regulatory activity as demonstrated by the rSNP within an enhancer at *NOS1AP* that elevates risk of cardiac arrhythmias [15]. Conversely, rSNPs may decrease regulatory activity as observed at *SH3TC2*, where the minor allele greatly reduces the binding ability of CREB [16]. Finally, an rSNP may create a novel TFBS as observed within the *TNF* promoter region, which results in the establishment of an OCT-1 binding site and increased gene expression [17]. While the cause of clinical variability within CMT disease is unknown, one possibility is that rSNPs, likely within TFBSs, may affect gene function in the cells of the peripheral nerve and exacerbate or alleviate the disease phenotype. Consistent with this notion, mutations in the promoter of *GJB1* cause CMT disease [18]. A subset of *GJB1* promoter mutations disrupt binding of the transcription factor SOX10, which is critical for Schwann cell development and function; SOX10 directly regulates genes important for Schwann cells and that have been implicated in demyelinating CMT disease (e.g., *MPZ*, *GJB1*, and *PMP22*) [19–21].

Here, we describe a genome-wide search for rSNPs with potential relevance to the peripheral nerve. We developed a computational pipeline to identify human non-coding genomic sequences that harbor a SNP with a major allele that is conserved among diverse vertebrate species: human, mouse, and chicken. Subsequently, we undertook a pilot functional evaluation of a subset of conserved genomic segments via luciferase assays in three cell lines relevant to the peripheral nerve and CMT disease: Schwann cells, motor neurons, and muscle cells. Finally, we deeply characterized one validated rSNP, demonstrating that it resides in a SOX10-responsive CRE that may regulate *Tubb2b*, which is mutated in patients with asymmetric polymicrogyria [22]. Interestingly, our results show that strict conservation analyses may not consistently enrich for CREs and associated rSNPs, thus underscoring the employment of complementary approaches.

## Results

### Genome-wide computational predictions of regulatory SNPs (rSNPs)

**Fig. 1:**
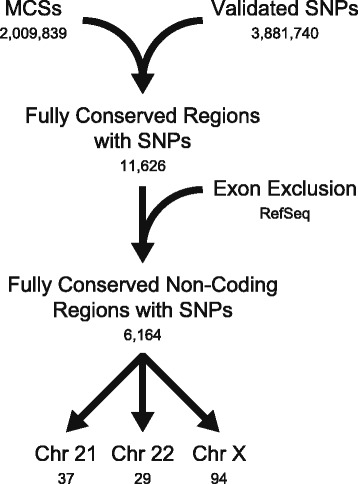
A computational pipeline to identify putative regulatory SNPs. The human, mouse, and chicken genomes were aligned, and genomic segments five base-pairs in length or greater and identical in all three species were identified to compile a panel of multiple-species conserved sequences (MCSs). Overlap between the MCS dataset and validated ‘by-frequency’ SNPs from db SNP130 was determined. Exons were excluded using RefSeq entries, and a pilot set of 160 regions were identified on chromosomes 21, 22, and X. The number of regions in each resulting dataset are indicated below the label

To identify and prioritize a set of putative CREs that harbor rSNPs, we developed a novel computational pipeline (Fig. 1). First, the human (hg18), mouse (mm9), and chicken (Gal3) genomes were downloaded from the UCSC Genome Browser [23], aligned using MultiPipMaker [24], and the alignments analyzed using ExactPlus [25] to identify genomic segments that are identical among the three species and at least five base pairs in length [26]. Next, conserved regions overlapping a SNP that was ‘validated by-frequency’ [i.e., SNPs that were submitted (dbSNP 130) with allele frequency information] were identified to generate a panel of conserved regions harboring a SNP. A similar analysis was performed using dbSNP 142 (Additional file 1: Figure S1). Finally, regions that overlapped exons (≥1 bp) were removed using the RefSeq database. The above process established a panel of 6197 SNPs that reside within 6164 conserved, non-coding sequences (Additional file 2).

### Identification of enhancers relevant to the peripheral nerve

To validate the efficacy of our computational approach, we selected a set of 160 regions (~ 2.5% of the total dataset; Additional file 3), which includes all genomic segments harboring a SNP that we identified on chromosome X (94 regions), chromosome 22 (29 regions), and chromosome 21 (37 regions). To functionally assess these 160 genomic segments, we tested each for the ability to direct luciferase reporter gene expression in vitro using immortalized cell lines relevant to the peripheral nerve: Schwann cells (S16), motor neurons (MN1), and muscle cells (myoblasts; C2C12). S16 cells are an immortalized rat Schwann cell line that expresses myelin-associated genes (e.g., *MAG*, *MPZ*, and *PMP22*) and transcription factors (e.g. SOX10 and EGR2) [27, 28]. MN1 cells were generated by somatic cell fusion between mouse spinal motor neurons and mouse neuroblastoma cells and exhibit traits similar to motor neurons including the ability to form neurite projections [29]. C2C12 cells are a mouse muscle progenitor cell line [30] that can be differentiated into muscle cells [31]. This process is incomplete, however, resulting in a heterogeneous population of cells and yielding transfection data that are difficult to interpret. Therefore, we focused our analyses on S16 and MN1 cell lines, but include the C2C12 data as supplemental material since they represent a critical tissue for peripheral nerve diseases. Briefly, each putative enhancer harboring the major SNP allele (Additional file 3) was cloned upstream of a minimal promoter directing luciferase gene expression [25]. Sequences surrounding each conserved region were selected based on the PhastCons 17-way vertebrate alignment dataset (mean = 885 bp, range = 105 bp to 2497 bp) [32]. The selected sequences were then separately transfected into each cell line, and luciferase activity was measured relative to a control vector with no genomic insert. Regions demonstrating a greater than five-fold increase in luciferase activity relative to the control vector were considered to have ‘strong’ activity and were selected for further analyses.

**Fig. 2:**
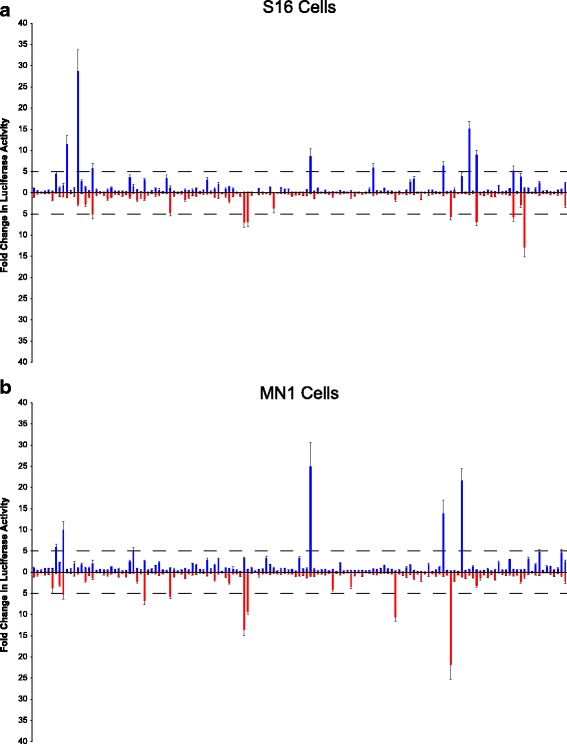
Activity of a pilot set of putative regulatory elements on chromosomes 21, 22, and X in Schwann cells and motor neurons. 144 genomic regions containing the major SNP allele were cloned upstream of a luciferase reporter gene and tested in the forward (blue bars) or reverse (red bars) orientation in S16 (**a**) and MN1 cells (**b**). The activity of each genomic segment is expressed relative to a control vector with no genomic insert (first bar in each frame). Dashed lines indicate a five-fold increase in activity over the control vector, and error bars show standard deviations

We successfully cloned 144 of the 160 genomic segments in our pilot study. Of the 16 regions lost: one SNP was removed in updated databases and was excluded from further analysis, six were amplified along with another region in the same PCR product, eight could not be amplified, and one could not be cloned. The regions were named SNP Conservation (“SC”) followed by the chromosome and were numbered from the p-arm to the q-arm. For example, SCX-1 is the most distal region identified on the p-arm of chromosome X. Each of the 144 putative enhancers was subjected to Sanger sequencing to verify the presence of the major allele and then transfected into S16, MN1, and C2C12 cell lines. The activity of each region was assessed in both the forward and reverse orientation with respect to the minimal promoter. Of the 144 regions tested in S16 cells, 13 demonstrated ‘strong’ luciferase activity in at least one orientation: SCX-3, SCX-4, SCX-21, SCX-39, SCX-58, SCX-60, SCX-65, SCX-67, SCX-78, SCX-81, SC21-13, SC21-16, and SC21-20 (Fig. 2a and Table 1). In experiments using MN1 cells, 11 of the 144 regions demonstrated ‘strong’ luciferase activity in at least one orientation: SCX-3, SCX-4, SCX-21, SCX-45, SCX-58, SCX-60, SCX-63, SC21-10, SC21-12, SC22-1, and SC22-8 (Fig. 2b and Table 1). In experiments using differentiated C2C12 cells, 21 of the 144 regions demonstrated ‘strong’ luciferase activity in at least one orientation: SC21-10, SC21-16, SC21-18, SC21-27, SC21-33, SC21-34, SC22-1, SC22-8, SC22-14, SCX-3, SCX-4, SCX-18, SCX-20, SCX-21, SCX-33, SCX-45, SCX-52, SCX-58, SCX-60, SCX-63, and SCX-67 (Additional file 4: Figure S2 and Table 1). In sum, we identified 28 unique regions with strong luciferase activity in at least one orientation in Schwann cells, motor neurons, and/or muscle cells. While we did not exclude conserved regions harboring multiple SNPs, no such element exceeded our threshold for activity in the above experiments.

**Table 1:** Twenty-eight genomic regions displayed regulatory activity in peripheral nerve relevant cells

Region	Fold-increase Forward^a^	Fold-increase Reverse^a^	Coordinates (hg18)	rs Number
SC21-10	5.91 (M) 8.10 (C)	0.25 (M) 0.33 (C)	chr21:21,313,182-21,313,188	rs7277262
SC21-12	9.95 (M)	5.34 (M)	chr21:22,535,874-22,535,880	rs2827297
SC21-13	11.42 (S)	1.04 (S)	chr21:27,318,695-27,318,702	rs233616
SC21-16	28.67 (S) 30.17 (C)	2.73 (S) 3.56 (C)	chr21:29,424,534-29,424,540	rs2832203
SC21-18	1.37 (C)	8.90 (C)	chr21:33,139,128-33,139,135	rs2833975
SC21-20	5.81 (S)	5.04 (S)	chr21:33,273,214-33,273,219	rs2834040
SC21-27	16.05 (C)	4.71 (C)	chr21:36,269,198-36,269,214	rs2835196
SC21-33	13.85 (C)	3.40 (C)	chr21:38,940,551-38,940,556	rs16996658
SC21-34	6.22 (C)	0.33 (C)	chr21:38,958,107-38,958,118	rs8130434
SC22-1	2.58 (M) 2.17 (C)	6.68 (M) 6.35 (C)	chr22:16,689,437-16,689,442	rs5992119
SC22-8	1.00 (M) 1.05 (C)	5.73 (M) 7.53 (C)	chr22:25,678,449-25,678,472	rs5761863
SC22-14	4.70 (C)	13.83 (C)	chr22:26,146,779-26,146,784	rs733164
SCX-3	0.20 (S) 3.34 (M) 3.21 (C)	6.97 (S) 13.56 (M) 10.79 (C)	chrX:15,529,186-15,529,192	rs4646115
SCX-4	0.17 (S) 0.67 (M) 0.86 (C)	6.85 (S) 9.25 (M) 8.31 (C)	chrX:17,730,099-17,730,104	rs2187846
SCX-18	14.59 (C)	0.98 (C)	chrX:31,252,044-31,252,049	rs7884417
SCX-20	7.39 (C)	1.59 (C)	chrX:31,435,143-31,435,149	rs3788892
SCX-21	8.63 (S) 25.00 (M) 6.60 (C)	0.18 (S) 0.84 (M) 0.75 (C)	chrX:31,764,702-31,764,707	rs1379871
SCX-33	0.67 (C)	13.92 (C)	chrX:85,443,058-85,443,064	rs6623642
SCX-39	5.84 (S)	0.50 (S)	chrX:86,429,198-86,429,205	rs16980794
SCX-45	0.37 (M) 0.31 (C)	10.62 (M) 5.49 (C)	chrX:92,656,893-92,656,900	rs12687113
SCX-52	0.50 (C)	5.02 (C)	chrX:99,454,087-99,454,093	rs7064056
SCX-58	6.33 (S) 13.83 (M) 14.69 (C)	0.64 (S) 0.93 (M) 1.56 (C)	chrX:121,682,472-121,682,478	rs17273301
SCX-60	0.43 (S) 0.41 (M) 0.50 (C)	5.73 (S) 21.81 (M) 8.86 (C)	chrX:123,382,405-123,382,410	rs2076164
SCX-63	21.68 (M) 12.81 (C)	1.04 (M) 0.42 (C)	chrX:125,247,437-125,247,470	rs16998722
SCX-65	15.05 (S)	0.41 (S)	chrX:125,925,884-125,925,893	rs5930055
SCX-67	8.97 (S) 0.56 (C)	6.90 (S) 8.91 (C)	chrX:127,229,700-127,229,720	rs17266605
SCX-78	5.21 (S)	5.80 (S)	chrX:146,960,885-146,960,891	rs6525876
SCX-81	1.09 (S)	12.85 (S)	chrX:147,430,625-147,430,635	rs17252118

^a^Activity only shown for cell line(s) where region was active

S = S16, M = MN1, C = C2C12

### The minor alleles of eight peripheral nerve enhancers significantly reduce regulatory activity

**Fig. 3:**
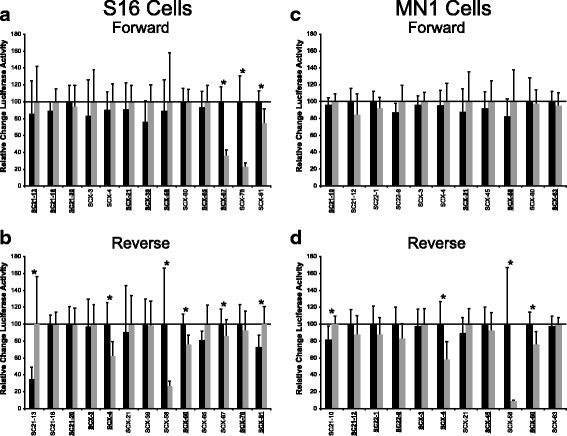
Eight genomic regions display allele-specific differences in regulatory activity in Schwann cells and motor neurons. **a** and **b** The activity of the major (black bars) and minor (grey bars) alleles of the 13 regions active in Schwann cells (Fig. 2) were evaluated in the forward (**a**) or reverse (**b**) orientation. **c** and **d** The major and minor alleles of the 11 genomic segments active in MN1 cells were compared as in panels a and b. In all panels, the allele with higher luciferase activity was set to “100”, error bars represent standard deviations, bold and underlined text indicate the orientation(s) that were active in the experiments shown in Fig. 2, and asterisks indicate a significant change in activity (*p* ≤ 0.05)

The 13 genomic segments active in Schwann cells, the 11 genomic segments active in motor neurons, and the 21 genomic segments active in muscle cells were mutagenized to the minor allele and reassessed in the respective cell line to test for allele-specific differences in regulatory activity. Each allele was tested in both orientations (forward and reverse, relative to the minimal promoter) regardless of the endogenous orientation with respect to the predicted target gene. In reporting these data, the more active allele of each region was set to ‘100%’, and the activity of the less active allele was expressed relative to the more active allele. In Schwann cells, SC21-13, SCX-4, SCX-58, SCX-60, SCX-67, SCX-78, and SCX-81 displayed allele-specific differences in luciferase activity (Fig. 3a, b, and Table 1). In motor neurons, SC21-10, SCX-4, SCX-58, and SCX-60 displayed allele-specific differences in luciferase activity (Fig. 3c, d, and Table 1). In C2C12 cells, SC21-18, SC21-27, SC22-8, SCX-4, SCX-21, SCX-45, and SCX-67 displayed allele-specific differences in luciferase activity (Additional file 5: Figure S3). Combined, we identified 13 unique regions displaying significant allele-specific differences in regulatory activity between the major and minor alleles. Three regions were unique to S16 cells, one region was unique to MN1 cells, and five regions were unique to C2C12 cells, while the remaining four regions demonstrated reduced regulatory activity in a combination of cells (Table 1).

### Predicting differential transcription factor binding to active regions

One possible explanation for the allele-specific differences described above is that the SNP alleles display differential affinity for transcription factors. To test this possibility, we used an in silico transcription factor binding site prediction program, TRANSFAC [33]. Sequence fragments of 30 base pairs centered around each major SNP allele were generated. The major allele was swapped for the minor allele and both sequences were submitted to TRANSFAC. The sequences were assessed for TFBS predictions using the TRANSFAC Match algorithm and the vertebrate database of transcription factors, minimizing the sum of false positive and false negative errors. Results were filtered to display only unique differences in predicted TFBSs, which may explain the differential activity.

**Fig. 4:**
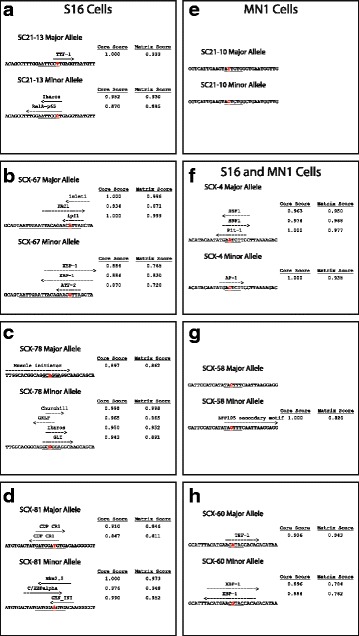
TRANSFAC predictions of transcription factor binding sites. TRANSFAC was used to assess for differential TFBS predictions between the major and minor alleles of SNP alleles that had a significant effect on luciferase activity. Results are shown for four regions active only in S16 cells [SC21-13 (**a**), SCX-67 (**b**), SCX-78 (**c**), and SCX-81 (**d**)], one region active only in MN1 cells [SC21-10 (**e**)], and three regions active in both cell types [SCX-4 (**f**), SCX-58 (**g**), and SCX-60 (**h**)]. Thirty base pairs surrounding the SNP alleles of each region were submitted to TRANSFAC. Dashed arrows indicate the position and direction of the predicted TFBS, the name of the transcription factor is indicated above each arrow, and the core and matrix scores are indicated at the right. Only allele-specific TFBS predictions are displayed. Underlined base pairs indicate conserved bases, and SNP alleles are highlighted in red and bold text

For the seven genomic segments differentially active in Schwann cells (Fig. 4a-d and f-h), each had at least one allele-specific TFBS prediction. Interestingly, none of the four Schwann cell specific regions [SC21-13, SCX-67, SCX-78, and SCX-81 (Fig. 4a-d)] harbored predicted TFBSs known to be important for these cells. While these results may be due to the limitations of TRANSFAC, they may also illustrate potentially novel roles of the predicted transcription factors in Schwann cells. For the four genomic segments differentially active in motor neurons (Fig. 4e-h), one (SC21-10) displayed no allele-specific predictions (Fig. 4e). This result may indicate that the SNP does not ablate or alter TFBS binding affinity or it may reflect the incomplete nature of TFBS databases. For the seven genomic segments differentially active in muscle cells (Additional file 6: Figure S4), each had at least one allele-specific TFBS prediction; two of these segments demonstrated regulatory activity only in muscle cells (SC21-18 and SC21-27). Strikingly, a LEF-1 binding site prediction is unique to the minor allele of SC21-27. *Lef-1* expression increases in mouse muscle cells following muscle injury [34]. Our data show that the minor allele of SC21-27 demonstrates significantly lower regulatory activity compared to the major allele (Additional file 5: Figure S3). Indeed, LEF-1 can act as a transcriptional repressor [35, 36] suggesting that the increased activity of the major allele is due to decreased LEF-1 binding; however, further study is needed to determine the role of the putative LEF-1 binding site in regulatory activity. Taken together, TRANSFAC revealed differential TFBS predictions across many of the genomic segments showing allele-specific differences in regulatory activity. While the significance of these predictions vary with respect to what is known about Schwann cell, motor neuron, and muscle cell biology, some may represent novel findings relevant to gene regulation in these cells.

### Identification of two rSNPs within SOX10 response elements

**Fig. 5:**
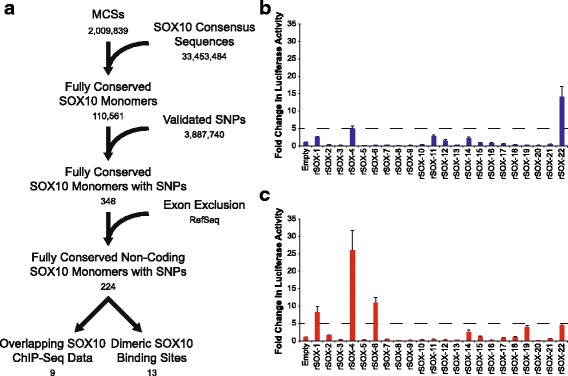
Identification of putative SOX10 response elements in Schwann cells. **a** All SOX10 consensus sequences in the human genome were identified, and these data were intersected with multiple-species conserved sequences (MCSs; see text for details). Overlap between the conserved SOX10 monomers and SNPs validated ‘by-frequency’ from dbSNP 130 was determined, and exons were excluded using RefSeq entries. This dataset of conserved, non-coding SOX10 monomers harboring a SNP was prioritized by identifying regions overlapping SOX10 ChIP-Seq data or by identifying a dimeric SOX10 consensus sequence. The number of regions in each resulting dataset is indicated under the label. **b** and **c** The 22 genomic segments from panel A were cloned upstream of a luciferase reporter gene and tested in the forward (**b**; blue bars) and reverse (**c**; red bars) orientation in SOX10-positive S16 cells. The activity of each genomic segment is expressed relative to a control vector with no genomic insert (‘Empty’ in **b** and **c**). Four regions displayed greater than five-fold activity (indicated by the dashed line) in at least one orientation. Error bars indicate standard deviations

Our pipeline successfully identified a small panel of putative rSNPs in a transcription-factor-independent approach. We next wanted to evaluate our computational predictions in the context of TFBS information (i.e., motif analysis and ChIP-Seq data). To this end, we evaluated the conserved regions, identified above, harboring a SNP from dbSNP 130 (6164 regions; Fig. 1 and Additional file 2) for the presence of a predicted SOX10 binding site (Fig. 5a). SOX10 was chosen because it is critical for Schwann cell function and regulates genes involved in peripheral nerve myelination [19–21]. SOX10 also directly binds to DNA via a well-defined consensus sequence as either a monomer or as a dimer when two monomeric sequences are oriented in a head-to-head fashion [37–39]. We first identified all SOX10 monomeric consensus sequences within the human genome [5′ to 3′: ACAAA, ACACA, ACAAT, or ACAAG (and the reverse complement of each)]. We then overlapped these data with the conserved regions harboring a SNP, which revealed 224 putative monomeric SOX10 binding sites containing a SNP and conserved among human, mouse, and chicken (Additional file 7). This dataset was further prioritized by only including sequences that overlap with SOX10 ChIP-Seq data [39] [resulting in nine regions, referred to as rSNP-containing and SOX10-relevant (rSOX) 1 through 9 in Fig. 5a and Table 2] and by separately assessing for the presence of a dimeric SOX10 consensus sequence where both monomers were conserved, but only one monomer was required to contain a SNP (resulting in 13 regions, referred to as rSOX-10 through 22 in Fig. 5a and Table 2).

**Table 2:** Twenty-two conserved SOX10 consensus sequences containing SNPs

Name	Conserved Region (hg18)	rs Number
rSOX-1	chr2:44834620-44834625	rs3738980
rSOX-2	chr3:62426974-62426979	rs6445273
rSOX-3	chr4:146183931-146183936	rs34577920
rSOX-4	chr6:22818474-22818479	rs16886790
rSOX-5	chr6:98577359-98577364	rs12524696
rSOX-6	chr6:98692222-98692227	rs17814604
rSOX-7	chr7:131601713-131601718	rs1364510
rSOX-8	chr13:66589464-66589469	rs17082112
rSOX-9	chr15:55214627-55214632	rs2703617
rSOX-10	chr1:48904340-48904354	rs1966247
rSOX-11	chr1:168449895-168449935	rs16863114
rSOX-12	chr6:118599910-118599921	rs17335828
rSOX-13	chr7:9853948-9853966	rs12702949
rSOX-14	chr7:95350538-95350566	rs10249566
rSOX-15	chr8:37281347-37281384	rs17333409
rSOX-16	chr8:138460036-138460047	rs16907090
rSOX-17	chr9:80243161-80243189	rs17788061
rSOX-18	chr10:78070796-78070831	rs17469556
rSOX-19	chr10:130596685-130596718	rs11819115
rSOX-20	chr11:31400150-31400183	rs1376362
rSOX-21	chr11:125354123-125354139	rs11607720
rSOX-22	chr16:53169712-53169728	rs1186802

rSOX-1 through rSOX-9 are conserved monomeric SOX10 consensus sequences that overlap with SOX10 ChIP-Seq data

rSOX-10 through rSOX-22 are conserved dimeric SOX10 consensus sequences

**Fig. 6:**
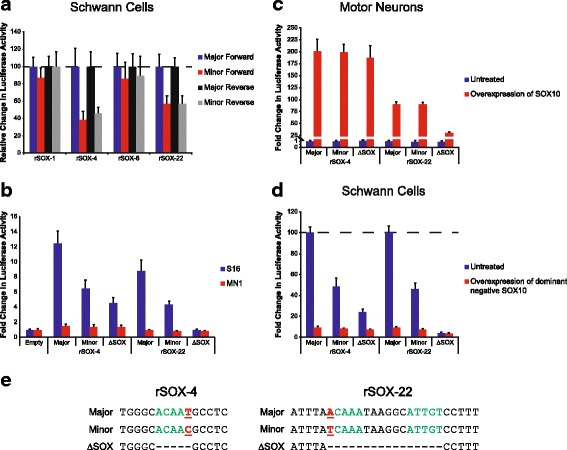
rSOX-4 and rSOX-22 harbor regulatory SNPs that alter the function of SOX10 consensus sequences. **a** Each allele of the four rSOX regions that displayed regulatory activity were assessed in both orientations in S16 cells. Bar colors indicate major allele in the forward orientation (blue), minor allele in the forward orientation (red), major allele in the reverse orientation (black), and minor allele in the reverse orientation (grey). For each orientation, the minor allele is expressed relative to the major allele. **b** Major, minor, and binding-site-deleted (ΔSOX) alleles of rSOX-4 and rSOX-22 were evaluated for regulatory activity in the more active orientation, in S16 (blue bars) or MN1 (red bars) cells. **c**-**d** Major, minor, and binding-site-deleted (ΔSOX alleles of rSOX-4 and rSOX-22 were evaluated for regulatory activity with and without a construct to express wild-type SOX10 in MN1 cells (**c**) or dominant-negative SOX10 in S16 cells (**d**). Data from untreated cells are in blue and data from cells co-transfected with a SOX10 expression construct are in red. In all panels, error bars indicate standard deviations. **e** Sequence variants studied within rSOX-4 and rSOX-22. The nucleotides surrounding each variant studied [major allele, minor allele, and deleted SOX10 binding site (∆SOX)] are shown. SOX10 monomeric sites unaffected by the SNP are displayed in green. The SNP within the SOX10 monomeric site is indicated by bold, underlined, and red text. Deleted nucleotides are indicated by dashes. Each variant name is displayed on the left and corresponds to the sequences tested in previous panels

The major allele of each of the 22 rSOX regions was cloned and evaluated for regulatory activity in S16 cells via luciferase assays as described above; SOX10 and SOX10 target genes are expressed in S16 cells [40]. Sequences surrounding each conserved rSOX region was selected for cloning based on the PhastCons 17-way vertebrate alignment dataset (mean = 729 bp, range = 319 bp to 1834 bp) [32]. These efforts revealed four genomic segments that exceeded a five-fold threshold of activity in at least one orientation: rSOX-1, rSOX-4, rSOX-6, and rSOX-22 (Fig. 5b and c, and Table 3). To assess for allele-specific differences in regulatory activity, the four genomic segments with strong activity were mutagenized to the minor allele and reassessed in luciferase assays in S16 cells. Two regions, rSOX-4 and rSOX-22, showed a significant difference in luciferase activity between the two alleles (Fig. 6a). The minor allele of rSOX-4 demonstrated 45.8% of the activity compared to the major allele, and the minor allele of rSOX-22 demonstrated 57.0% of the activity of the major allele.

**Table 3:** Four genomic segments harboring a SOX10 consensus sequence display regulatory activity in Schwann cells

Region	Fold-increase Forward	Fold-increase Reverse	Coordinates (hg18)^a^	rs Number	MAF^b^
rSOX-1	2.57	8.29	chr2:44834620-44834625	rs3738980	0.3035
rSOX-4	4.83	25.96	chr6:22818474-22818479	rs16886790	0.2001
rSOX-6	0.33	11.01	chr6:98692222-98692227	rs17814604	0.1014
rSOX-22	14.12	4.44	chr16:53169712-53169728	rs1186802	0.4219

^a^Coordinates for the conserved SOX10 monomeric sites for rSOX-1, −4, and − 6 and the dimeric site for rSOX-22

^b^MAF is the minor allele frequency from the 1000 Genomes Project [72]

We further assessed the role of SOX10 in regulating rSOX-4 and rSOX-22 by deleting the putative SOX10 binding sites harboring a SNP (a single monomeric sequence for rSOX-4 and a single dimeric sequence for rSOX-22) and performing luciferase activity assays in S16 cells. In each case we observed an even more dramatic decrease in regulatory activity upon deleting the SOX10 binding site. Relative to the major alleles, rSOX-4 ΔSOX demonstrated 36.5% activity, and rSOX-22 ΔSOX demonstrated 10.9% activity (Fig. 6b). We also tested each SNP allele and ΔSOX10 genomic segment for activity in MN1 cells, which do not express endogenous SOX10. Neither region harboring the major allele showed strong activity in MN1 cells, consistent with SOX10 being required for the observed regulatory activities (Fig. 6b). Furthermore, neither the minor SNP allele nor deletion of the SOX10 consensus sequence had an effect on regulatory activity in SOX10-negative MN1 cells.

We next evaluated the role of SOX10 in regulating rSOX-4 and rSOX-22 by studying the activity of each allele in SOX10-negative MN1 cells co-transfected with a construct to overexpress wild-type SOX10. In the absence of SOX10, neither allele of the two regions demonstrated activity in MN1 cells, consistent with the results above. In contrast, in the presence of SOX10, both regions displayed strong activity, approximately 200-fold and 89-fold higher for rSOX-4 and rSOX-22, respectively (Fig. 6c). Conversely, when we performed luciferase assays with a construct to express a dominant-negative SOX10 protein (E189X) to deplete endogenous SOX10 function in S16 cells [41], both regions displayed severely decreased activity (~ 9%) compared to the respective major alleles (Fig. 6d). Taken together, our data indicate that SOX10 is both necessary and sufficient for the in vitro regulatory activity of rSOX-4 and rSOX-22 in cultured Schwann cells.

### Ablation of rSOX-4 in cultured Schwann cells reduces Tubb2b expression

**Fig. 7:**
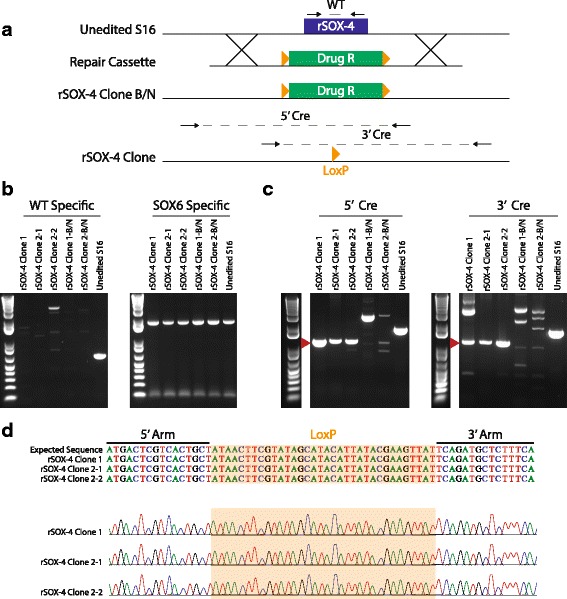
CRISPR-mediated deletion of rSOX-4 in S16 cells. **a** A cartoon depiction of the rSOX-4 deletion strategy is shown. The 651 bp rSOX-4 enhancer in unedited cultured rat Schwann (S16) cells is indicated by a blue rectangle. The drug resistance repair cassette is indicated by a green rectangle and the loxP sequences by orange triangles. Cross lines represent homologous regions for recombination-mediated repair during CRISPR/Cas9-mediated mutagenesis. Arrows represent the diagnostic PCR primers used in panels B and C, with the name of the corresponding PCR product [wild-type (WT), 5’ Cre, and 3’ Cre] above. **b** Wild-type (WT) specific PCR was performed on the three Cre:GFP-positive rSOX-4 clonal cell lines (Clone 1, Clone 2-1, Clone 2-2), the parental, pre Cre:GFP transfection (Clone1 – B/N and Clone 2 – B/N) cell lines, and unedited S16 cells. A SOX6-specific PCR was performed (right panel) as a DNA positive control. **c** Diagnostic PCR was performed across the 5′ (left panel) and 3′ (right panel) recombination sites for the same samples indicated in panel B using external (genomically anchored) and internal (repair template anchored) primers. The red arrowhead indicates the expected size for a single loxP scar. **d** Sequencing results from the rSOX-4 clonal cell lines in panel C (red arrowhead). The expected sequence was generated in silico based on proper recombination, Cre excision, and presence of a loxP scar

Identifying the target gene(s) of *cis*-acting regulatory elements is essential for understanding the biological significance of rSNPs that reside within these elements. To identify the target gene of an enhancer/rSNP pair identified in this study, we selected rSOX-4 and deleted it from S16 cells using CRISPR/Cas9 [42] technology. We then performed RNA-Seq analysis in an unbiased attempt to identify candidate target genes with altered expression. The rSOX-4 element is a 651-base-pair region that resides in a ~ 1 Mb interval devoid of annotated genes (Additional file 8: Figure S5A) and that displayed genomic features indicating that it is an active, SOX10-bound enhancer, including: H3K27 acetylation from adult rat peripheral nerve [43], SOX10 ChIP-Seq from P15 rat sciatic nerve [39], and DNase hypersensitivity from the S16 cell line [26] (Additional file 8: Figure S5A). Briefly, repair templates were generated with ~ 1 Kb arms of homology to the 5′ and 3′ regions surrounding the 651-base-pair rSOX-4 element, and flanking a floxed blasticidin (or neomycin) resistance cassette (Fig. 7a). S16 cells were transfected with the blasticidin repair template, human codon optimized Cas9 (hCas9), and one of two gRNAs targeting rSOX-4 (Additional file 9: Figure S6). Next, cells were selected for stable and proper integration of the blasticidin repair template and flow sorted to generate clonal cell lines. We generated two clones, one using gRNA-1 (rSOX-4 Clone 1-B) and one using gRNA-2 (rSOX-4 Clone 2-B) that had proper integration of the blasticidin resistance repair cassette. Integration was assessed using a diagnostic PCR with one primer outside the targeting vector and one primer within the drug repair template. These PCR products were sequence verified to ensure the integrity of the genomic DNA. This process was repeated once more using the neomycin resistance template to generate double-resistant clonal cell lines with no wild-type alleles at rSOX-4 (Fig. 7b). Clones were then transiently transfected with a Cre:GFP expression construct to remove the drug resistance cassettes, and GFP-positive cells were flow sorted to generate clonal populations. A final diagnostic PCR was performed to assess for complete removal of the drug resistance cassettes, and the products were sequence verified to ensure that only a single loxP scar remained (Fig. 7c and d). We generated three clonal cell lines: two share a common parental cell line prior to Cre:GFP transfection (rSOX-4 Clone 2-1 and 2-2) while the other was independently generated (rSOX-4 Clone 1).

**Fig. 8:**
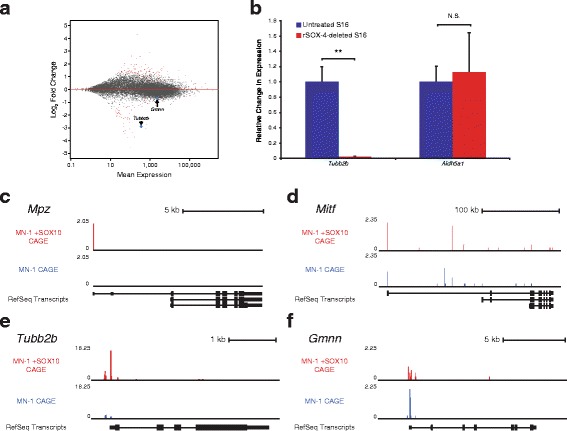
rSOX-4 regulates expression of *Tubb2b.* **a** MA plot demonstrating differential gene expression between the rSOX-4 deleted cells and untreated S16 cells. Each dot represents a gene and red dots indicate a significant difference in expression between the two cell populations (adjusted *p* < 0.05). Genes with a positive or negative log2 fold change demonstrated higher or lower expression in the rSOX-4 cells line compared to the untreated S16 cells, respectively. *Tubb2b* and *Gmnn* are indicated by arrows. **b** Digital droplet PCR (ddPCR) was performed to validate RNA-Seq findings for *Tubb2b*. Data from the untreated cells are shown by the blue bars, and data from rSOX-4-deleted cells are shown by the red bars. *Aldh5a1* was used as a control gene, which showed no expression changes between the two cell populations. **c-f** SOX10 increases *Tubb2b* expression in heterologous cells. MN1 cells were transfected with a construct to express GFP-SOX10, sorted into GFP-positive and GFP-negative populations, and subjected to cap analysis gene expression (CAGE). CAGE reads mapping to the *Mpz* (**c**), *Mitf* (**d**), *Tubb2b* (**e**), and *Gmnn* (**f**) loci were normalized to reads per million and are indicated in red (SOX10-positive cells) and blue (SOX10-negative cells). RefSeq-annotated transcripts at each locus in the mouse genome (mm10) are shown in black and, in each case, are transcribed from left to right

We performed RNA-Seq on the clonal cells described above to assess for gene expression changes. Briefly, RNA was extracted from the three rSOX-4 deleted cell lines and from two unmodified S16 cell populations (one was the parental cell for all three rSOX-4 clonal cell lines). RNA was subjected to 50-bp, single-end read analysis, and reads were aligned to the rat (rn5) genome using STAR [44] and counted using HTSeq [45]. We then used DESeq2 [46] to identify genes that were differentially expressed between the rSOX-4 mutant and unmodified S16 cells. This revealed 198 genes significantly differentially expressed between the two cell populations (Fig. 8a). Of the 198 genes, six were on the same chromosome (chr 17) as rSOX-4, and only two of these demonstrated decreased expression in rSOX-4 mutant S16 cells relative to unmodified S16 cells: *Tubb2b* and *Gmnn* (Table 4). We focused on these two because of our luciferase data suggesting these regions act as enhancers. To validate these findings in an independent experimental system, we performed digital droplet PCR (ddPCR) to quantify *Tubb2b* and *Gmnn* expression in rSOX-4 mutant S16 cells and unmodified S16 cells. We validated the decrease in expression of *Tubb2b* using ddPCR (Fig. 8b), but these studies did not reveal a decrease in *Gmnn* expression (data not shown) as observed in our RNA-Seq analysis. Thus, decreased *Gmnn* expression may have been an artifact of the RNA-Seq experiments. To account for gene expression changes due to clonal expansion, we also isolated RNA from five clonal cell populations generated from unmodified S16 cells, performed RNA-Seq as described above, and reanalyzed for differential gene expression between the rSOX-4 mutant S16 cells and all unmodified S16 cell lines. In agreement with the ddPCR validation studies, *Tubb2b*, but not *Gmnn*, was significantly decreased in expression in the rSOX-4 mutant cells compared to all unmodified S16 samples (Additional file 10: Figure S7). Finally, we performed capped analysis gene expression (CAGE) [47] on MN1 cells with and without a construct to over-express SOX10 (see Methods for details; Fogarty and Antonellis, manuscript in preparation). In the presence of SOX10, the known SOX10 target genes *Mpz* and *Mitf* show increased reads per million (Fig. 8c and d). Importantly, in the presence of SOX10, *Tubb2b* shows a dramatic increase in expression while *Gmnn* does not. Taken together, these results support the conclusion that *Tubb2b* is regulated by SOX10 activity at rSOX-4.

**Table 4:** Six genes on chromosome 17 are differentially expressed in Schwann cells deleted for rSOX-4

Gene	*p*-value^a^	Fold Change^b^	Distance from rSOX-4^c^
*Tubb2b*	1.48E-10	−7.41	8.9
*Rbm24*	0.025	2.63	23
*Gmnn*	0.042	−1.61	1.9
*Etl4*	0.044	1.73	47
*Mylip*	0.044	1.59	20.5
*Akr1c19*	0.045	2.50	29

^a^Benjamini-Hochberg adjusted p-value

^b^Fold change calculated relative to unmodified S16 cells (negative values indicate lower expression in rSOX-4 mutant cells, positive values indicate higher expression in rSOX-4 mutant cells)

^c^Distances are given in million basepairs

### rSOX-4 directs *LacZ* expression in SOX10-positive cells at E11.5

To determine the physiological relevance of rSOX-4 in vivo, we assessed the ability of the genomic segment harboring the major allele to direct *LacZ* expression during mouse development. Because the regulatory activity of rSOX-4 is dependent on SOX10, we anticipated that rSOX-4 would demonstrate activity in tissues in which SOX10 is expressed. Based on the RNA-Seq data indicating that *Tubb2b* is the target gene of this enhancer, we selected embryonic day 11.5 (E11.5) due to the known role of *Tubb2b* in neuronal migration [22]. We reasoned that rSOX-4 and *Tubb2b* may be performing a similar role in migratory neural crest cells and neural crest derivatives; Schwann cells are derived from the neural crest. By E11.5 in mice, the neural tube has closed, and neural crest cells and derivatives have started to migrate [48–51].

**Fig. 9:**
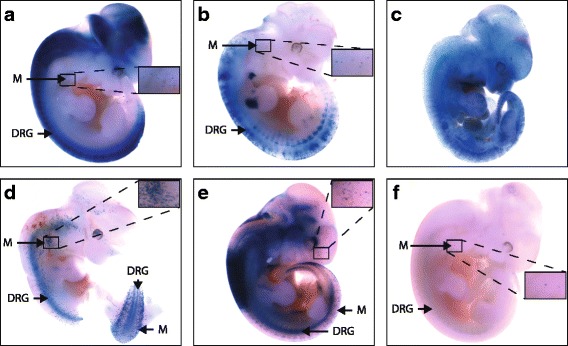
rSOX-4 directs in vivo enhancer activity during mouse development. Six transient transgenic mouse embryos were generated to harbor a transgene with rSOX-4 directing *LacZ* expression (**a**-**f**). Mice were sacrificed at E11.5, and expression patterns were determined by visual examination under a stereoscope (M = melanoblasts and DRG = dorsal root ganglia). Image cutouts show an enlarged section to demonstrate melanoblast expression

We cloned rSOX-4 upstream of the minimal *Hsp68* promoter directing *LacZ* expression [52] in the reverse orientation because this was the more active orientation in luciferase assays. The rSOX-4:*LacZ* transgene was liberated from the plasmid backbone, gel purified, and injected into mouse zygotes. Zygotes were implanted into pseudopregnant female mice, and embryos were allowed to develop until E11.5 and then harvested to examine *LacZ* expression. We isolated 54 embryos from ten mice and identified six LacZ positive mice (Fig. 9a-f). Five embryos demonstrated LacZ staining in the dorsal root ganglia and migrating melanoblasts (Fig. 9a, b, and d-f), both tissues in which SOX10 is expressed at E11.5 [53, 54]. A single embryo displayed ubiquitous blue LacZ staining throughout the entire embryo, likely a consequence of position effect (Fig. 9c). These experiments demonstrate that rSOX-4 is active in SOX10-positive cells during mouse development and suggest that *Tubb2b* plays an important role in neural crest cell function.

## Discussion

The identification and characterization of regulatory variation (e.g., rSNPs) will improve our understanding of human biology and disease. To study regulatory variation in the human genome we developed a computational pipeline to identify genomic sequences that harbor a SNP and that are conserved among human, mouse, and chicken. We functionally evaluated a pilot set of 144 conserved genomic segments in luciferase assays in three cell lines relevant to the peripheral nerve: Schwann cells, motor neurons, and muscle cells. We identified 28 (19% of the 144 elements) putative enhancers, including 13 (9% of the 144 elements) SNPs that demonstrated significant allele-specific differences in regulatory activity. We further assessed our pipeline by separately incorporating consensus sequence information and ChIP-Seq data for SOX10—a transcription factor essential for Schwann cells. We prioritized 22 conserved genomic segments harboring a SNP predicted to disrupt a SOX10 binding site and identified two (9% of the 22 elements) rSNPs with a significant effect on regulatory activity. Finally, we deeply characterized one rSNP-containing SOX10 response element by deleting the region in cultured Schwann cells to identify dysregulated genes and by generating transient transgenic mice to study in vivo enhancer activity. Our findings revealed a far-upstream regulatory variant that effects SOX10 function at *Tubb2b*, thus displaying the ability of our approach to find biologically relevant transcriptional regulatory elements and associated rSNPs.

The computational and functional studies described here yielded a large panel of SNPs that reside in highly conserved regions, RNA-Seq data from cultured Schwann cells, and small panels of regulatory SNPs (rSNPs) and *cis*-regulatory elements (CREs) useful for investigators studying peripheral nerve and SOX10 biology. Furthermore, the pipeline developed here is flexible and may accommodate additional CRE information when it becomes available; for example, we combined our pipeline with previously unavailable SOX10 ChIP-Seq [39], S16 DNase hypersensitivity [26], and H3K27 acetylation [43] datasets to prioritize putative SOX10 binding sites for functional validation. There are, however, several inherent limitations to our approach that could be improved upon. First, our computational predictions of rSNPs relied on non-coding sequence conservation among diverse vertebrate species. This limitation is problematic because rSNPs may reside in less well-conserved genomic regions [55]. Indeed, our efforts suggest that the employment of strict conservation analyses does not dramatically enrich for functional regulatory elements and associated rSNPs. To address this problem, one could overlap available SOX10 ChIP-Seq data [39] with our data set of SOX10 consensus sequences [26] and study SNPs that map to overlapping genomic sequences, regardless of sequence conservation. Second, we tested a small subset of the 6164 regions predicted by our computational approach. Recent advances in massively parallel reporter assays would allow for functional interpretation of most, if not all, of our predictions [56, 57]. The use of a minimally active promoter also restricts our interpretation because it only allows for detection of major alleles which direct high levels of reporter activity; using an active basal promoter could enable the identification of repressive elements. Finally, the rSNPs identified here were studied outside of the proper genomic context (i.e., a small genomic segment surround each SNP allele was studied for regulatory activity in a vector containing a luciferase reporter gene). Interestingly, the majority of the genomic segments and rSNPs that we studied showed orientation-specific activity and an orientation-specific effect of the rSNP on regulatory activity. With respect to the orientation-specific effects, while the classical definition of an enhancer is that it should act in an orientation-independent manner, this likely depends on the specific sequence of the element and how it is cloned into a reporter gene vector; indeed, enhancers with orientation-dependent activity in vitro and in vivo have been reported [58, 59]. With respect to the orientation-specific effect of the rSNP, one possible explanation for this is that the position of the rSNP in the reporter gene construct has orientation-specific effects on transcription factor and transcriptional machinery binding in vitro. While one genomic segment harboring an rSNP (rSOX-4) was evaluated via CRISPR-mediated deletion of the regulatory element, future analysis of rSNPs important for peripheral nerve biology and disease should include studying allele-specific effects at the endogenous locus in an appropriate cell or animal model.

Characterization of rSOX-4 via CRISPR/Cas9 [42] mediated deletion revealed *Tubb2b* as a strong candidate target gene of this enhancer. TUBB2B is a critical component of microtubules, and mutations in human *TUBB2B* result in polymicrogyria [22]. Interestingly, our transgenic mouse studies revealed that rSOX-4 directs *LacZ* expression in the dorsal root ganglia and migratory melanoblasts. A mouse strain harboring a GFP transgene reporter inserted into the endogenous *Tubb2b* locus has been generated [60]. The earliest time point the authors looked at was E14.5, which is after melanoblast migration has completed [61]; however, the expression patterns are strikingly similar to our transgenic mouse, as the authors observe GFP expression within the presumptive spinal cord, in the head and brain region, and in the dorsal root ganglia. There is no known role for TUBB2B in the neural crest or, more specifically, in Schwann cells; however, *Tubb2b* expression is highest in migratory neurons [22, 60]. Since rSOX-4 directed *LacZ* expression in migratory melanoblasts, this suggests that *Tubb2b* has a similar, uncharacterized role in cell migration in melanoblasts and other neural crest derived cells. Indeed, Schwann cells have a migratory phase before myelination [62].

Our RNA-Seq, CAGE, and in vitro and in vivo reporter gene data revealed *Tubb2b* as the most promising candidate target locus of the rSOX-4 enhancer. However, rSOX-4 resides nearly 9 Mb from *Tubb2b* and the largest distance reported between a regulatory element and a target transcriptional unit is currently 1.7 Mb [63]. Given the complex domain structure of nuclear organization, it is feasible that rSOX-4 regulates *Tubb2b*, but confirming this will require chromosome conformation capture experiments; until those experiments are complete, other potential target genes of rSOX-4 should be explored. For example, a recent release of the human genome (GRCh38) revealed that rSOX-4 resides within the first intron of a long non-coding RNA (lncRNA), LOC105374972. There is no known function of LOC105374972, but it has been classified as ‘validated’. After converting the coordinates of this lncRNA from human to rat, we were unable to detect any RNA-Seq reads corresponding to the lncRNA in our S16 data. This may be due to the generation of a polyA-selected RNA library for RNA-Seq; LOC105374972 may not be polyadenylated [64]. Another possible explanation for the absence of RNA-Seq reads is that LOC105374972 may not be expressed in Schwann cells. Additional studies are required to elucidate any functional role of the lncRNA LOC105374972 in SOX10-positive cells and to determine if it is regulated by rSOX-4.

## Conclusions

In this study, we developed a computational and functional pipeline that identified a small panel of *cis*-acting regulatory elements harboring regulatory SNPs (rSNPs). This pipeline revealed rSNPs in both a transcription factor independent and dependent manner. While our computational approach may be less relevant for biological systems with well-developed genomic data sets, we believe it allows a less-biased strategy for identifying rSNPs important for developmental stages and tissues for which ChIP-Seq and related genomic analyses are not feasible. We then deeply characterized one enhancer that is regulated by SOX10 (rSOX-4) through CRISPR-mediated deletion and in vivo transient transgenic mouse reporter experiments. Through these studies, *Tubb2b* was identified as a strong candidate target gene of rSOX-4. While additional experiments are necessary to determine the role of rSOX-4 in SOX10-positive cells, the rSNP within rSOX-4, the rSOX-4 enhancer, and the *Tubb2b* locus all represent excellent candidate modifiers of neurocristopathies. Additionally, understanding and characterizing the role of *Tubb2b* in neural crest-derived cells—in particular the dorsal root ganglia and melanoblasts—will provide insight into the biology of these cell populations.

## Methods

### PCR and cloning of putative regulatory elements

PCR was performed utilizing primers containing attB1 and attB2 Gateway cloning (Invitrogen) sequences to amplify a region surrounding each of the 144 conserved, non-coding regions with SNPs from human DNA (Additional file 3) and the 22 SOX10 regions (Table 2). The total amplified region was based on general conservation using the PhastCons 17-way vertebrate alignment [32]. If a region fell within a PhastCons track, the primers were designed to amplify the entire region defined by the track. If a region did not reside within a PhastCons track, then primers were designed to amplify a region conserved between human and mouse only (~ 500 bp). Each amplified region was BP cloned into pDONR221 using the Gateway cloning technology (Invitrogen). The regions were sequenced to verify the SNP allele and absence of additional mutations. These regions were subsequently LR cloned (Invitrogen) upstream of a minimal E1B promoter directing luciferase expression [25] in both the forward (pE1B Forward) and reverse (pE1B Reverse) orientations. Active regions were converted to the alternative allele, or SOX10 binding sites were deleted using site-directed mutagenesis. Mutagenic primers (sequences available upon request) were designed, and mutagenesis was performed on the regions cloned into pDONR221 using the QuikChange II Mutagenesis Kit (Agilent Technologies). The resulting constructs were sequenced to verify specificity and then LR cloned into pE1B as described above.

### Cell culture and transfection for luciferase experiments

We obtained immortalized rat Schwann (S16) cells in 2006 from Richard Quarles (NIH/NINDS) who originally established these cells [27]. We obtained mouse MN1 cells in 2004 from Kurt Fischbeck (NIH/NINDS), who obtained them directly from the laboratory that generated these cells (H. Kim, University of Chicago) [29]. The C2C12 [30] cells were obtained from ATCC (CRL-1772) in 2011. We routinely test our cells for mycoplasma contamination. All cell lines were verified for authenticity using gene expression experiments including RT-PCR and sequencing of products (all three cell lines), RNA-Seq (S16 cells), and CAGE (MN1 cells). S16 and MN1 cells were cultured at 37 °C in 5% CO_2_ in Dulbecco’s Modified Eagle Medium (DMEM; Invitrogen) containing 10% fetal bovine serum (FBS; Invitrogen), 1X glutamine (Gibco), and 1X Penicillin/Streptavidin (ThermoFisher). S16 and MN1 cells were plated at 10,000 cells/well of a 96-well plate. The cells were transfected the following day using Lipofectamine 2000 (Life Sciences). Each well was transfected with 200 ng of pE1B [25] plasmid containing the region of interest and 2 ng of a renilla expression construct. Cells were harvested 48 h after transfection and assessed using the Dual-Luciferase Reporter system (Promega). Luciferase activity was normalized to renilla activity, and all regions of interest were compared to a control vector with no human genomic insert (‘Empty’ in figures). For transcription factor overexpression assays in S16 and MN1 cells, an additional 100 ng of the overexpression plasmid was added per well. C2C12 cells were assessed as described above with the following changes [65]: they were plated at a concentration of 5000 cells/well in a 96-well plate, and 24 h after transfection the media was changed to differentiation media: Dulbecco’s Modified Eagle Medium (DMEM; Invitrogen) supplemented with 5% horse serum (FBS; Invitrogen), 1X glutamine (Gibco), and 1X Penicillin/Streptavidin (ThermoFisher). For all luciferase assays, at least eight technical replicates across three biological replicates (*n* = 24) were performed.

### TRANSFAC analysis

The major allele of each SNP was centered within 30 bp of genomic sequence and extracted using the UCSC Human Genome Browser. The minor allele nucleotide of the candidate rSNP was substituted into the above sequence to generate the minor allele sequence. The major and minor allele sequences were then submitted to TRANSFAC using the Match tool [66]; the ‘vertebrate, non-redundant’ and ‘minimize the sum of false positive and negative error rates’ options were used. Results were visually examined and manually filtered to exclude any predicted binding sites that were identical between the major and minor allele, regardless of the core or matrix scores.

### CRISPR-mediated deletion of rSOX-4

Two guide RNAs (gRNAs) were designed and cloned into a gRNA expression plasmid (Addgene plasmid #41824) as previously described [42]. The repair template was constructed using Gibson assembly [67] and cloned into pUC19. The 5′ and 3′ homology arms were PCR-amplified from S16 genomic DNA, the blasticidin resistance cassette was PCR amplified from pCMV/Bsd (ThermoFisher - Cat no. V510-20), and the neomycin resistance cassette was PCR amplified from the hCas9 backbone (Addgene plasmid #41815) [42]. Wild-type S16 cells were cultured as described above and plated at 100,000 cells/well in a 6-well dish 24 h prior to transfection. The cells were transfected with 3 μg of total DNA [1 μg of hCas9, 1 μg of gRNA expression plasmid, and 1 μg of linearized repair template (~ 1:1:1 M ratio)] using Lipofectamine 2000. Standard growth media was replaced after 4 h of transfection, and the cells were grown for 72 h at 37 °C and 5% CO_2_. The growth media was removed, and selection media (standard growth media with 4 μg/mL of blasticidin and/or 700 μg/mL of neomycin) was added. After selection (~ 3 days for blasticidin and ~ 14 days for neomycin) the cells were expanded to a T-75 flask before flow sorting into a 96-well plate to generate clonal populations. The cells were gradually expanded over ~ 1 month, genomic DNA was harvested, and diagnostic PCR was performed using genomic- and drug-specific primers. The procedure was repeated to target any remaining wild-type alleles using a neomycin resistance repair template; however, the gRNAs had to be reversed due to indels in the remaining wild-type alleles after blasticidin selection (Additional file 9: Figure S6; i.e. gRNA1 was used in rSOX-4 Clone 2-B and gRNA2 was used in rSOX-4 Clone 1-B). Additionally, we were unable to amplify the blasticidin repair template from rSOX-4 Clone 2 following the second round of clone generation. Double-resistant clones (blasticidin and neomycin) were expanded in T-75 flasks, transiently transfected with a Cre:GFP expression plasmid (Addgene plasmid #13776) to remove the floxed drug cassettes, and flow sorted to generate clonal GFP-positive cells.

### RNA-Seq analysis of wild-type and rSOX-4 mutant cells

RNA was isolated from the three rSOX-4 mutant S16 lines, two unmodified S16 lines [one parental cell line of the rSOX-4 mutants (passage 9) and one older passage (passage 39)], and five clonal expansions of unmodified S16 cells using TRIzol extraction (ThermoFisher). mRNA libraries were generated using TruSeq (Illumina) and subjected to 50 bp single-end sequencing on a HiSeq2000. Two technical replicates of the rSOX-4 mutant and two unmodified S16 lines cells were pooled and run across two sequencing lanes which resulted in ~ 21.5 million reads per cell line. The five clonally expanded S16 cells were only sequenced once. Read quality was assessed using FastQC (http://www.bioinformatics.babraham.ac.uk/projects/fastqc/) and reads were aligned to the rat Rnor_5.0 (Ensembl) reference genome using STAR [44]. Default parameters were used, except only uniquely mapped reads were allowed (~ 82% of total reads mapped uniquely). HTSeq [45] was used to count the number of reads per gene using default parameters with the stranded reads function disabled. Finally, differential gene expression between the rSOX-4 mutant and unmodified S16 cells was determined using DESeq2 [46]. All programs were run on the ARCTS flux servers at the University of Michigan. An identical analysis for the five clonally expanded unmodified S16 cells was performed as described above. Data for the two technical and two biological replicates of unmodified S16 cells are located on NCBI GEO (GSE81709).

### Digital droplet PCR

cDNA was synthesized from RNA extracted from the rSOX-4 mutant and unmodified S16 cells using the High-Capacity cDNA Reverse Transcription Kit (Applied Biosystems). Each reaction contained 3.2 μL ultrapure water, 2 μL 10X random primers, 2 μL 10X RT buffer, 1 μL RNase inhibitor, 0.8 μL dNTPs, 1 μL RNA in 10 μL of water, and 1 μL MultiScribe reverse transcriptase. The reaction was incubated for 10 min at 25 °C, then 2 h at 37 °C, then 85 °C for 5 s, and finally held at 4 °C. The resulting cDNA was diluted 1:333 in ultrapure water prior to the digital droplet PCR (ddPCR). Each ddPCR contained 11.5 μL 2X ddPCR supermix (BioRad), 1 μL FAM probe, 1 μL HEX probe, 6 μL cDNA template (diluted 1:333), and 3.5 μL water. FAM probes were designed against the gene of interest while HEX probes were against the control gene (*Gapdh*). Probes and primers were ordered from IDT PrimeTime as predesigned assays for rat except for the FAM probe for *Aldh5a1*, which was custom designed using the IDT PrimerQuest tool with default parameters for two primers and one probe. ddPCRs were set up and run per the manufacturer’s instructions (BioRad), and results were analyzed using QuantaSoft software to determine the absolute quantification of each gene of interest. Significant differences between the rSOX 4-mutant and unmodified S16 cells was performed using a Student’s T-test. Each cDNA sample was assessed in four technical replicates, and the average was used in subsequent analyses. For rSOX-4 mutant cells, one cDNA sample was used for each mutant line, and all three were combined for the final analysis. For unmodified S16 cells, three independent biological replicates were assessed and combined for the final analysis.

### Cap analysis gene expression (CAGE)

The human SOX10 open-reading frame in pDONR221 (Addgene plasmid #24749) was obtained and SOX10 was cloned into pcDNA-DEST53 using LR Clonase (ThermoFisher) to generate a GFP-SOX10 expression construct. MN1 cells [29] were plated at a density of 100,000 cells per mL and transfected with the GFP-SOX10 expression construct using Lipofectamine 2000 (ThermoFisher). After 48 h, cells were harvested, suspended in PBS, and sorted into GFP-positive and GFP-negative populations using a SY3200 Cell Sorter (Sony). RNA was isolated from each cell population using the RNeasy Mini Kit (QIAGEN). A CAGE library was generated for each sample as described [47], with modifications made for compatibility with current generation Illumina sequencing platforms. Each library was sequenced on an Illumina MiSeq (4 million reads generated per library). The sequencing data was analyzed using FastQC (http://www.bioinformatics.babraham.ac.uk/projects/fastqc), filtered using TagDust2 [68], mapped using BWA [69], clustered using Paraclu [70], and visualized using BEDtools [71] and the UCSC Genome Browser [23].

### Generation of transient transgenic mice harboring rSOX-4: *LacZ*

rSOX-4 was LR cloned upstream of a *LacZ* reporter gene directed by the *HSP68* minimal promoter [52]. The plasmid was sequence verified, and the rSOX-4:*LacZ* transgene fragment was excised from the vector backbone by digestion with *Sal*I. The transgene was isolated and injected into mouse zygotes by the University of Michigan transgenic animal core, and zygotes were implanted into pseudopregnant mice. Pregnant female mice were sacrificed (CO_2_ vapor was employed because it is easy to use and is the recommended choice of the Panel of the American Veterinarian Medical Association) at embryonic day 11.5 (E11.5), and embryos were dissected and placed into 15 mL of ice cold fixative solution: 0.2% glutaraldehyde and 2% formaldehyde in PBS. Embryos were fixed for 20 min on ice and washed three times for 10 min with wash buffer: 2 mM MgCl_2_, 5 mM EGTA, and 0.02% NP40 in PBS. After washing, embryos were transferred to a 50 mL conical vial containing ~ 25 mL of LacZ staining solution: 5 mM potassium ferrocyanide, 5 mM potassium ferricyanide, and 1 mg/mL X-gal (Invitrogen) in wash buffer; X-gal was diluted to 50 mg/mL in dimethylformamide prior to addition to the staining solution. Embryos were gently rocked at 37 °C overnight in the dark. Embryos were then washed three times for 10 min each with wash buffer and imaged using a dissecting scope with QImaging camera (QICAM FAST1394) and software (Qcapture Pro Version 6).

## Additional files

Additional file 1: **Figure S1.** A computational pipeline to identify putative regulatory SNPs. The human, mouse, and chicken genomes were aligned, and genomic segments that are five base-pairs in length or greater and identical in all three species were identified to compile a panel of multiple-species conserved sequences (MCSs). Overlap between the MCS dataset and validated ‘by-frequency’ SNPs from dbSNP 142 was determined, and exons were excluded using RefSeq entries. The number of regions in each resulting dataset are indicated below the label. (AI 1127 kb)

Additional file 2: List of the 6197 SNPs residing within 6164 conserved, non-coding sequences. Columns 1 through 3 are BED file formatted hg18 coordinates of the conserved, non-coding regions. Columns 4 through 6 are BED file formatted hg18 coordinates of the SNPs residing within the identified regions. The final column is the rs ID number for each SNP. (TXT 352 kb)

Additional file 3: List of the pilot set of regions and SNPs from chromosome 21, 22, and X. Columns 1 through 3 are BED file formatted hg18 coordinates of the conserved, non-coding regions. Columns 4 through 6 are BED file formatted hg18 coordinates of the SNPs residing within the identified regions. The final column is the rs ID number for each SNP. (TXT 9 kb)

Additional file 4: **Figure S2.** Activity of a pilot set of putative regulatory elements on chromosomes 21, 22, and X in muscle (C2C12) cells. 144 genomic regions containing the major SNP allele were cloned upstream of a luciferase reporter gene and tested in the forward (blue bars; upper) or reverse (red bars; lower) orientation in C2C12 cells. The activity of each genomic segment is expressed relative to a control vector with no insert (first column) set to “1”. Dashed lines indicate a five-fold increase in activity over the control vector, and error bars show standard deviations. (AI 1152 kb)

Additional file 5: **Figure S3.** Seven regions display allele-specific differences in luciferase activity in muscle (C2C12) cells. (A) The activity of the major (black bars) and minor (grey bars) alleles of the 21 regions active in muscle cells (Additional file 4: Figure S2) were evaluated in the forward (A) or reverse (B) orientation. In both panels, the allele with higher luciferase activity was set to “100”, error bars represent standard deviations, bold and underlined text indicate the orientation(s) that were active in experiments shown in Additional file 4: Figure S2, and asterisks indicate a significant change in activity (*p* ≤ 0.05). (AI 1169 kb)

Additional file 6: **Figure S4.** TRANSFAC predictions of transcription factor binding sites within elements active in muscle cells. TRANSFAC was used to assess for allele-specific TFBS predictions between the major and minor alleles of SNP alleles that had a significant effect on luciferase activity. Results are shown for seven regions active in C2C12 cells: SC21-18 (A), SC21-27 (B), SC22-8 (C), SCX-4 (D), SCX-21 (E), SCX-45 (F), and SCX-67 (G). Thirty base-pairs surrounding each SNP allele was submitted to TRANSFAC. Dashed arrows indicate the position and direction of the predicted TFBS, the name of the transcription factor is indicated above each arrow, and the core and matrix scores are indicated at the right. Only allele-specific TFBS predictions are displayed. Underlined base pairs indicate conserved bases, and the SNP alleles are highlighted in red and bold text. (AI 1331 kb)

Additional file 7: List of the 224 SOX10 consensus sites harboring SNPs. Columns 1 through 3 are BED file formatted hg18 coordinates of the conserved, non-coding SOX10 consensus sites. Columns 4 through 6 are BED file formatted hg18 coordinates of the SNPs residing within the identified regions. The final column is the rs ID number for each SNP. (TXT 14 kb)

Additional file 8: **Figure S5.** rSOX-4 overlaps genomic features associated with enhancers. (A; Top) rSOX-4 (red box) overlaps histone 3 lysine 27 acetylation peaks (H3K27Ac; purple track), SOX10 ChIP-Seq peaks from rat sciatic nerve (pink track), and S16 DNase hypersensitivity peaks (S16 DNase HSS; black track). (A; Bottom) Zoomed-out browser from above to show surrounding rn5 RefSeq genes. Dashed lines to the green bar indicate the position of rSOX-4 within a gene desert. (B; Top) rSOX-22 (red box) does not overlap any genomic feature assessed. (B; Bottom) Zoomed-out browser from above to show surrounding rn5 RefSeq genes. Dashed lines to the green bar indicate the position of rSOX-22 within a gene desert. In all panels, track names are at the left, the scale for each track is indicated, and the width of each browser window is noted at the top (Kb = kilobase pairs and Mb = megabase pairs). (AI 1851 kb)

Additional file 9: **Figure S6.** CRISPR cuts all alleles in S16 cells. A wild-type specific diagnostic PCR was performed on rSOX-4 Clone 1-B and 2-B, and PCR products were sequenced. (A) A single base pair insertion (yellow box) of an adenine was identified in the remaining non-recombined alleles of rSOX-4 Clone 1-B that resides within the gRNA-1 recognition site. (B) A 79 base-pair deletion was detected encompassing the gRNA-2 cut site in rSOX-4 Clone 2-B. The yellow box represents the expected 5′ sequence, and the blue box represents the 3′ sequence. In both panels, the rn5 genome sequence is the expected sequence, and gRNA-1, SOX10 consensus site, and gRNA-2 are labeled and indicated by lines under the nucleotide sequences. (AI 1471 kb)

Additional file 10: **Figure S7.** *Tubb2b* expression is significantly reduced in rSOX-4 mutant S16 cells. An MA plot of the mean expression of every gene (dots) against the log2-fold change is shown. The mean expression is calculated as the mean of the normalized counts across all samples, and the log2 fold change is relative to unmodified S16 cells. Genes above the red line (“0”) indicate higher expression in rSOX-4 mutant cells, and genes below the red line indicate lower expression in rSOX-4 mutant cells. Red dots indicate genes significantly differentially expressed between rSOX-4 mutant, and unmodified S16 cells (*p* < 0.05). *Gmnn* and *Tubb2b* are labeled and indicated by arrows. (AI 17314 kb)

## Abbreviations

CAGE: Cap Analysis of Gene Expression
ChIP-Seq: Chromatin Immunoprecipitation Sequencing
CMT: Charcot-Marie-Tooth Disease
CRE: *cis*-acting regulatory element
ddPCR: Digital Droplet polymerase chain reaction
GWAS: Genome-Wide Association Studies
MAF: Minor Allele Frequency
rSNP: Regulatory Single Nucleotide Polymorphism
rSOX: Regulatory single nucleotide polymorphism contained within a conserved SOX10 consensus site
SC: SNP Conservation
SNP: Single Nucleotide Polymorphism
TFBS: Transcription factor binding site
